# Comprehensive Transcriptome Sequencing of Tanaidacea with Proteomic Evidences for Their Silk

**DOI:** 10.1093/gbe/evab281

**Published:** 2021-12-14

**Authors:** Keiichi Kakui, James F Fleming, Masaru Mori, Yoshihiro Fujiwara, Kazuharu Arakawa

**Affiliations:** 1 Faculty of Science, Hokkaido University, Sapporo, Hokkaido, Japan; 2 Institute for Advanced Biosciences, Keio University, Tsuruoka, Yamagata, Japan; 3 Graduate School of Media and Governance, Keio University, Fujisawa, Kanagawa, Japan; 4 Research Institute for Global Change, Japan Agency for Marine-Earth Science and Technology (JAMSTEC), Yokosuka, Kanagawa, Japan; 5 Faculty of Environment and Information Studies, Keio University, Fujisawa, Kanagawa, Japan

**Keywords:** tanaidaceans, transcriptome, silk, proteome, phylogenomics

## Abstract

Tanaidaceans are small benthic crustaceans that mainly inhabit diverse marine environments, and they comprise one of the most diverse and abundant macrofaunal groups in the deep sea. Tanaidacea is one of the most thread-dependent taxa in the Crustacea, constructing tubes, spun with their silk, for shelter. In this work, we sequenced and assembled the comprehensive transcriptome of 23 tanaidaceans encompassing 14 families and 4 superfamilies of Tanaidacea, and performed silk proteomics of *Zeuxo ezoensis* to search for its silk genes. As a result, we identified two families of silk proteins that are conserved across the four superfamilies. The long and repetitive nature of these silk genes resembles that of other silk-producing organisms, and the two families of proteins are similar in composition to silkworm and caddisworm fibroins, respectively. Moreover, the amino acid composition of the repetitive motifs of tanaidacean silk tends to be more hydrophilic, and therefore could be a useful resource in studying their unique adaptation of silk use in a marine environment. The availability of comprehensive transcriptome data in these taxa, coupled with proteomic evidence of their silk genes, will facilitate evolutionary and ecological studies.

Significance

Here we report the transcriptome sequencing and assembly of 23 tanaidaceans, a group of small benthic crustaceans, encompassing 14 families and 4 superfamilies of Tanaidacea. This is the largest data set of this underrepresented crustacean order to date. Comprehensive transcriptomic data will accelerate tanaidacean studies in evo-devo contexts, an otherwise untouched field. Tanaidaceans are unique in their use of silk to create tube-shaped habitat, and we combined the transcriptome assembly with proteomic analysis of the silk protein to characterize two families of tanaidacean silk genes. The knowledge of the molecular compositions of tanaidacean silks spun in sea water would substantially contribute to the study of water-resistant protein materials, taking advantage of the renewable, biodegradable nature of these silks.

## Introduction

The order Tanaidacea is a group of benthic crustaceans with about 1,500 described species (Anderson 2020). Tanaidaceans are small, typically a few millimeters long, and mainly inhabit marine environments ranging in depth from intertidal to hadal zones. They often occur in shallow and deep waters at high densities (e.g., more than 10,000 individuals/m^2^; Larsen 2005) and comprise one of the most diverse and abundant macrofaunal groups in the deep sea (Larsen et al. 2015). Tanaidaceans show diverse modes of life, including burrowers, empty-gastropod-shell carriers like hermit crabs, and inhabitants in tubes constructed with threads or mucus (hereafter known as, “tube dwellers”) (Kakui 2016).

Tanaidacea is one of the most thread-dependent taxa in the Crustacea and the diversity of its tube dwelling species is high, including numerous species in the superfamily Paratanaoidea, which contains more than half of all known tanaidacean species. Tanaidoidea are almost exclusively tube dwellers (Larsen et al. 2015); and a few tube-dwelling species have also been reported in Apseudoidea (Kakui and Hiruta 2017), but never reported within another extant superfamily, Neotanaoidea. Three different thread/mucus-producing systems are currently known: the thoracic (in Paratanaoidea and Tanaidoidea), pereopodal (in Kalliapseudidae of Apseudoidea), and pleotelsonal (in Parapseudidae of Apseudoidea) gland systems (Kakui and Hiruta 2014, 2017; Kaji et al. 2016). For tanaidaceans, tubes have many advantages: They provide stable shelter, space for copulation, and nurseries (e.g., Johnson and Attramadal 1982a, 1982b), and tube dwellers expand tanaidacean habitats to nonseafloor environments, such as the surfaces of marine vertebrates (e.g., sea turtles; Tanabe et al. 2017). The mucus and silk also provide a substrate on which other materials (mineral grains, shell debris, algae, organic detritus, sponge spicules, etc.) are adhered for further protection. The acquisition of thread-producing capabilities must thus have played an important role in tanaidacean evolution and diversification.

Various arthropod lineages have evolved to utilize silk for critical purposes, including foraging, nesting, mating, metamorphosis, and communication (Sutherland et al. 2010). There is substantial interest in the utility of these protein materials in industrial applications (Abascal and Regan 2018; Numata 2020), which have been intensively studied within silkworms and spiders due to their silk’s renewable, biodegradable, and thus sustainable nature, as well as their extraordinary mechanical properties. Water and moisture sensitivity is a key issue in such applications, and thus the properties of silks spun in water have been extensively studied in caddisfly larvae (Insecta: Trichoptera) (Lane et al. 2015; Wang et al. 2015; Ashton et al. 2016; Ashton and Stewart 2019). Alongside freshwater caddisworm silk, the knowledge of the molecular compositions of tanaidacean silks spun in sea water would substantially contribute to the study of water-resistant protein materials.

To this end, here we report the comprehensive transcriptome sequencing and assembly of 23 tanaidaceans encompassing 14 families and 4 superfamilies of Tanaidacea, which, to the best of our knowledge, is the largest data set of this underrepresented crustacean order. Moreover, we provide proteomic evidence, in the characterization of at least two families of silk proteins.

## Results

A high-quality transcriptome assembly of 22 tanaid taxa and one outgroup (R07, *Asellus hilgendorfii*, Isopoda) was successfully obtained, mostly with BUSCO completeness above 90% (table 1). One sample had low amounts of extracted RNA which resulted in incomplete transcriptome assembly (66.2%, R05 *Apseudomorpha* sp.), and this sample was omitted from subsequent analyses. These samples encompass 14 families and 4 superfamilies of Tanaidacea.

**Table 1:** Summary of Samples and Assembly Statistics Used in This Study @

No.	Taxon	Family	Superfamily or Higher	Sex	Thread Use	Number of Transcripts	Longest Transcript	N50 Length	BUSCO Completeness	BUSCO Completeness (Longest Isoform)
R02	*Zeuxo ezoensis*	Tanaididae	Tanaidoidea, Tanaidomorpha	Not checked	Observed by KK (thoracic-gland system)	41,001	30,563	3,040	C: 92.5% [S: 40.0%, D: 52.5%], F: 1.1%, M: 6.4%, *n*: 1,013	C: 90.3% [S: 73.2%, D: 17.1%], F: 1.4%, M: 8.3%, *n*: 1,103
R04	*Sinelobus* sp.	Tanaididae	Tanaidoidea, Tanaidomorpha	Male	Observed by KK (thoracic-gland system)	38,989	27,117	2,886	C: 92.3% [S: 33.4%, D: 58.9%], F: 1.2%, M: 6.5%, *n*: 1,013	C: 91.5% [S: 52.7%, D: 38.8%], F:1.0%, M: 7.5%, *n*: 1,103
R05	*Apseudomorpha* sp.	Metapseudidae	Apseudoidea, Apseudomorpha	Female	Not observed by KK (epibenthic)	35,946	14,971	1,370	C: 66.2% [S: 45.5%, D: 20.7%], F: 11.8%, M: 22.0%, *n*: 1,013	C: 64.6% [S: 61.3%, D: 3.3%], F:9.4%, M: 26.0%, *n*: 1,103
R06	*Paradoxapseudes littoralis*	Apseudidae	Apseudoidea, Apseudomorpha	Hermaphrodite	Not observed by KK (burrower)	37,127	25,608	1,785	C: 77.6% [S: 61.5%, D: 16.1%], F: 8.2%, M: 14.2%, *n*: 1,013	C: 76.6% [S: 75.9%, D: 0.7%], F:5.4%, M: 18.0%, *n*: 1,103
R07	*Asellus hilgendorfii*	Asellidae	Isopoda	Male	Not observed by KK (epibenthic)	43,376	28,641	3,220	C: 93.0% [S: 48.4%, D: 44.6%], F: 1.5%, M: 5.5%, *n*: 1,013	C: 89.2% [S: 88.3%, D: 0.9%], F:0.7%, M: 10.1%, *n*: 1,103
R08	*Apseudes* sp.	Apseudidae	Apseudoidea, Apseudomorpha	Hermaphrodite	Not observed by KK (burrower)	35,766	28,835	2,817	C: 93.9% [S: 75.3%, D: 18.6%], F: 1.3%, M: 4.8%, *n*: 1,013	C: 91.8% [S: 91.5%, D: 0.3%], F:1.2%, M: 7.0%, *n*: 1,103
R10	*Hexapleomera sasuke*	Tanaididae	Tanaidoidea, Tanaidomorpha	Female	Observed by KK (thoracic-gland system)	39,120	24,047	2,662	C: 92.5% [S: 49.4%, D: 43.1%], F: 1.0%, M: 6.5%, *n*: 1,013	C: 91.4% [S: 87.6%, D: 3.8%], F:1.7%, M: 6.9%, *n*: 1,103
R11	*Phoxokalliapseudes tomiokaensis*	Kalliapseudidae	Apseudoidea, Apseudomorpha	Female	Observed by KK (pereopodal-gland system)	40,894	37,262	2,951	C: 93.6% S: 63.4%, D: 30.2%], F:0.8%, M: 5.6%, *n*: 1,103	C: 89.1% [S: 88.5%, D: 0.6%], F:0.6%, M: 10.3%, *n*: 1,103
R12	*Carpoapseudes spinigena*	Apseudidae	Apseudoidea, Apseudomorpha	Female	Not observed by KK (burrower)	42,133	27,246	2,472	C: 89.1% [S: 64.3%, D: 24.8%], F:3.1%, M: 7.8%, *n*: 1,103	C: 83.9% [S: 83.2%, D: 0.7%], F:2.5%, M: 13.6%, *n*: 1,103
R14	*Pseudosphyrapus quintolongus*	Sphyrapodidae	Apseudoidea, Apseudomorpha	Not checked	Not observed in one congeneric species by KK (burrower)	41,538	29,339	2,741	C: 94.1% [S: 58.1%, D: 36.0%], F:0.5%, M: 5.4%, *n*: 1,103	C: 92.5% [S: 91.7%, D: 0.8%], F:0.8%, M: 6.7%, *n*: 1,103
R15	*Pakistanapseudes* sp.	Parapseudidae	Apseudoidea, Apseudomorpha	Female	nd	47,279	28,137	2,743	C: 94.2% [S: 52.3%, D: 41.9%], F:1.6%, M: 4.2%, *n*: 1,103	C: 92.6% [S: 91.4%, D: 1.2%], F:1.2%, M: 6.2%, *n*: 1,103
R18	*Arctotanais alascensis*	Tanaididae	Tanaidoidea, Tanaidomorpha	Not checked	Observed by KK (thoracic-gland system)	36,930	26,503	2,412	C: 89.9% [S: 62.0%, D: 27.9%], F:2.0%, M: 8.1%, *n*: 1,103	C: 87.2% [S: 85.8%, D: 1.4%], F:2.9%, M: 9.9%, *n*: 1,103
R19	*Neotanais* cf. *kuroshio*	Neotanaidae	Neotanaoidea, Tanaidomorpha	Female	nd	36,954	24,195	1,995	C: 79.5% [S: 66.3%, D: 13.2%], F:6.6%, M: 13.9%, *n*: 1,103	C: 78.1% [S: 77.2%, D: 0.9%], F:4.8%, M: 17.1%, *n*: 1,103
R20	*Chauliopleona* cf. *sinusa*	Akanthophoreidae	Paratanaoidea, Tanaidomorpha	Not checked	nd	42,339	26,566	3,028	C: 92.0% [S: 48.6%, D: 43.4%], F:1.4%, M: 6.6%, *n*: 1,103	C: 90.6% [S: 89.7%, D: 0.9%], F:1.3%, M: 8.1%, *n*: 1,103
R21	*Agathotanais misakiensis*	Agathotanaidae	Paratanaoidea, Tanaidomorpha	Not checked	One congeneric species in a tube was reported once	37,541	26,527	2,768	C: 91.6% [S: 71.8%, D: 19.8%], F:1.6%, M: 6.8%, *n*: 1,103	C: 89.9% [S: 89.3%, D: 0.6%], F:1.7%, M: 8.4%, *n*: 1,103
R22	*Tanaella kommritzia*	Tanaellidae	Paratanaoidea, Tanaidomorpha	Not checked	Individuals in a tube were observed by KK (thoracic-gland system)	36,514	26,731	2,619	C: 91.7% [S: 63.6%, D: 28.1%], F:1.0%, M: 7.3%, *n*: 1,103	C: 90.0% [S: 89.4%, D: 0.6%], F:1.1%, M: 8.9%, *n*: 1,103
R23	*Parapseudes algicola*	Parapseudidae	Apseudoidea, Apseudomorpha	Not checked	Observed by KK (pleotelsonal-gland system)	37,032	28,519	3,000	C: 94.2% [S: 75.1%, D: 19.1%], F:0.8%, M: 5.0%, *n*: 1,103	C: 92.5% [S: 92.0%, D: 0.5%], F:0.7%, M: 6.8%, *n*: 1,103
R24	*Heterotanoides* sp.	Heterotanoididae	Paratanaoidea, Tanaidomorpha	Female	nd	42,874	43,880	2,295	C: 86.0% [S: 47.5%, D: 38.5%], F:2.9%, M: 11.1%, *n*: 1,103	C: 80.6% [S: 79.6%, D: 1.0%], F:5.2%, M: 14.2%, *n*: 1,103
R27	*Chondrochelia* sp.	Leptocheliidae	Paratanaoidea, Tanaidomorpha	Female	Congeneric species in a tube were reported	38,274	27,116	2,043	C: 85.6% [S: 72.2%, D: 13.4%], F:4.8%, M: 9.6%, *n*: 1,103	C: 75.6% [S: 75.1%, D: 0.5%], F:2.0%, M: 22.4%, *n*: 1,103
R32	*Siphonolabrum* sp.	Anarthruridae	Paratanaoidea, Tanaidomorpha	Female	Confamilial species in a tube were reported	40,128	24,313	3,118	C: 91.1% [S: 54.9%, D: 36.2%], F:1.8%, M: 7.1%, *n*: 1,103	C: 88.5% [S: 88.1%, D: 0.4%], F:2.4%, M: 9.1%, *n*: 1,103
R33	*Tanaopsis* sp.	Tanaopsidae	Paratanaoidea, Tanaidomorpha	Female	One congeneric species in a tube was reported once	36,398	27,345	2,526	C: 91.0% [S: 69.9%, D: 21.1%], F:2.0%, M: 7.0%, *n*: 1,103	C: 90.0% [S: 89.4%, D: 0.6%], F:1.8%, M: 8.2%, *n*: 1,103
R34	*Paranarthrura* sp.	Agathotanaidae	Paratanaoidea, Tanaidomorpha	Female	One congeneric species in a tube was reported once	43,123	24,746	2,928	C: 92.3% [S: 46.0%, D: 46.3%], F:1.0%, M: 6.7%, *n*: 1,103	C: 90.5% [S: 90.0%, D: 0.5%], F:0.9%, M: 8.6%, *n*: 1,103
R35	*Parakanthophoreus* sp.	Akanthophoreidae	Paratanaoidea, Tanaidomorpha	Not checked	nd	41,697	24,350	3,060	C: 91.2% [S: 45.2%, D: 46.0%], F:1.9%, M: 6.9%, *n*: 1,103	C: 90.2% [S: 89.7%, D: 0.5%], F:2.0%, M: 7.8%, *n*: 1,103
R36	*Akanthophoreus* sp.	Akanthophoreidae	Paratanaoidea, Tanaidomorpha	Female	nd	41,168	25,306	2,888	C: 91.8% [S: 53.2%, D: 38.6%], F:1.9%, M: 6.3%, *n*: 1,103	C: 90.8% [S: 90.0%, D: 0.8%], F:1.9%, M: 7.3%, *n*: 1,103

**Fig. 1.**
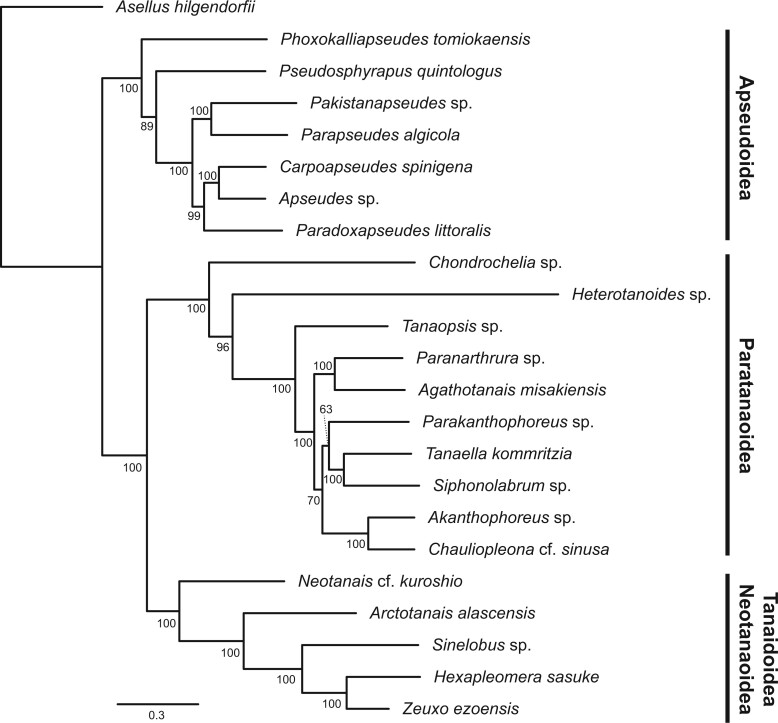
Phylogenetic tree of Tanaidacea sequenced in this work constructed with partition maximum likelihood method with 28 BUSCO genes. The tree was constructed in IQTree under the LG+F+I+G4 model. A total of 1,000 bootstraps were run, and bootstrap support values are marked on each node.

Our BUSCO phylogenetic analyses of Tanaidacea produced interesting and controversial results. Both the partitioned and unpartitioned maximum likelihood and the Bayesian analyses recovered the same topology with a very high level of support, suggesting a strong and reliable signal from the conserved BUSCO genes (fig. 1, supplementary figs. S1–S3, Supplementary Material online). Prior 18S analyses of the clade have previously recovered similar high-level clade structures (Kakui et al. 2011), with the Tanaidoidea and Neotanaoidea as sister taxa to the exclusion of Paratanaoidea, with Apseudoidea as the sister group to all other tanaidaceans. However, this is in contrast to a more classical understanding of the clade (Lang 1956; Lauterbach 1970; Gardiner 1975). This can be seen as a corroboration of our current molecular understanding of the order. However, the organization of taxa within the superfamilies differs from prior work. Notably, we find a monophyletic Apseudoidea inclusive of Kalliapseudidae under both maximum likelihood and Bayesian frameworks, which was inconsistently recovered in prior assessment of 18S genes, where it was not recovered under Parsimony or Minimum-Evolution (Kakui et al. 2011). Within the Paratanaoidea, our most noteworthy find is that, with increased sampling from within the clade, we do not recover the Akanthophoreidae as monophyletic, instead finding *Parakanthophoreus* in a monophyletic clade with *Tanaella kommritzia* and *Siphonolabrum* sp. to the exclusion of the two other akanthophoreids in the data set, *Akanthophoreus* sp. and *Chauliopleona* cf. *sinusa*. This discovery may prompt a review of the defining characteristics of the family in the future, though further genomic data from within Paratanaoidea is certainly required to explore these questions further.

**Fig. 2.**
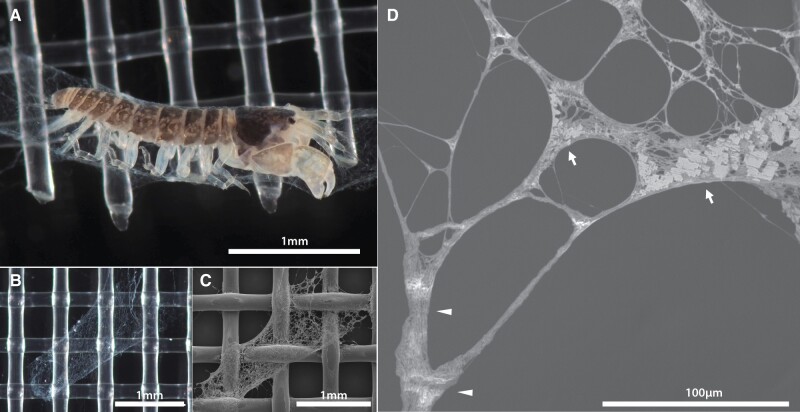
Microscope observation of *Z. ezoensis* silk. (*A*) *Z. ezoensis* in the tube constructed with its silk (Nylon mesh in the background). Tube observed in (*B*) light microscope and (*C*) scanning electron microscopy (SEM). (*D*) Close-up of the detailed structure of silk constituting the tunnel. Local structure is quite diverse, such as large fiber bundles (arrowhead) and nano-scale fibers with sheet like composites (arrow).

Detailed microscope observation of the silk tube of *Zeuxoezoensis* showed a complex organization of the tube (fig. 2). Nano-scale fibers form fine meshes, and were sometimes bundled into thick stems (fig. 2*D*, arrowhead). Moreover, the holes of fine meshes were often padded with sheet-like components (fig. 2*D*, arrow), which could be contaminants but could also suggest the use of nonsilk materials as a composite.

**Fig. 3.**
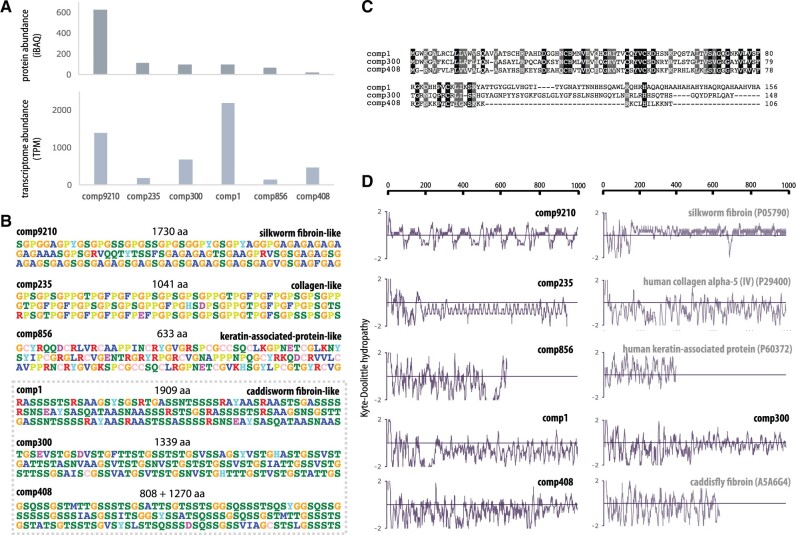
Proteomic analysis of *Z. ezoensis* silk. (*A*) Proteome and transcriptome abundances of six proteins detected in the proteome analysis. Comp9210 is the most abundant constituent of the silk tube. (*B*) Repetitive motifs of the six proteins. Comp9210 contains GAGAGS motif resembling the silkworm (GA)n. Comp235 has GPX motif resembling collagen, comp856 has CC motif resembling keratin-associated protein, and comp1, comp300, and comp408 resembles in the abundance of S of caddisworm fibroins. (*C*) Alignment of N-terminus of comp1, comp300, and comp408. High sequence conservation suggests the homology of these three proteins. (*D*) Kyte–Doolittle hydropathy plot of N-terminal 1,000 amino acids of the six proteins as well as their analogs. Positive value represents hydrophobicity.

**Fig. 4.**
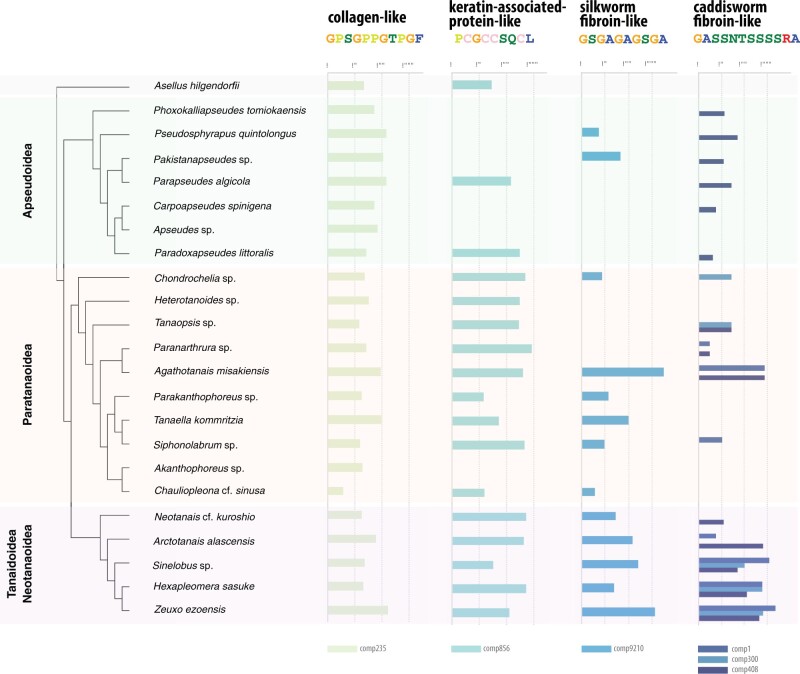
Conservation and expression levels of silk-constituting proteins. Bars represent the highest expression value of conserved transcript in each species in TPM. Orthologous transcripts comp1, comp300, and comp408 are grouped in “caddisworm fibroin-like” protein. Collagen-like comp235 and keratin-associated protein-like comp856 are conserved in the outgroup *A. hilgendorfii*, suggesting that these proteins are not the main silk constituents but rather a possible contamination of abundant protein in the mucus.

Proteomic analysis of the *Z. ezoensis* silk tube detected 60 proteins consistently in the five replicates, of which 41 did not match any of the proteins in the UniProt database. Excluding proteins with only one peptide support, high variability in abundance among replicates, and proteins without repetitive motifs, 21 transcripts (including isoforms) remained, that were further clustered into six proteins based on sequence identity (fig. 3). Screening and error correction of the nanopore long reads of cDNA successfully recovered the full-length sequence of five proteins, and N-terminal and C-terminal fragments of one protein (comp408). Of these 6 proteins, comp9210 was the most abundant protein (fig. 3*A*), and in fact it is the most abundant protein among the all 60 proteins detected, suggesting this protein to be the main component of the *Z. ezoensis* silk. Interestingly, all six of these proteins were large proteins (633–1,909 amino acids), and were mostly comprised characteristic repeats, which strongly suggests that all of these are structural proteins (fig. 3*B*). Comp9210 contains abundant (GA)n and GAGAGS resembling the fibroin of the silkworm *Bombyx mori*, GPG/GPY linker motif resembling the amorphous region of major ampullate spidroin (MaSp) of spiders, and another characteristic linker motif SGRVQQTYTSSF. N/C-terminal sequences do not show similarity to *B. mori* fibroin or MaSp, suggesting that these genes, whilst similar to the repetitive motifs, are not homologous. The repetitive motif of GPX of comp235, but not the terminal sequences, matches vertebrate collagen alpha-1 (*e*-value = 3e-57, XP_038241255), and likewise, the repetitive motif of CC of comp856 weakly matches keratin-associated protein (*e*-value = 8e-16, XP_018008435). The remaining three proteins (comp1, comp300, comp408) do not have significant matches, but all contain Serine-enriched motifs, which resemble the SXSXSXSX motif of caddisworm fibroin. Interestingly, although the repetitive motifs of these three proteins are similar but distinct, the N-terminal sequences are conserved (fig. 3*C*), suggesting that these three genes are orthologous. All of these six proteins show lower Kyle–Doolitle hydropathy values compared with the similar proteins, suggesting adaptation to underwater spinning of these silks. Searching through the 23 transcriptome assembly, the conservation and gene expression abundance of four classes of proteins (collagen-like, keratin-associated protein-like, silkworm fibroin-like, and caddisworm fibroin-like) were assessed (fig. 4). All of these proteins were mostly conserved throughout Tanaidacea, and collagen-like and keratin associated protein-like proteins (comp235 and comp856) were even conserved outside of Tanaidacea, in the outgroup Isopoda *A. hilgendorfii* which does not utilize silk. Therefore, these proteins are likely contaminants from the mucus as abundant structural proteins, and the main silk constituents are most likely to be the silkworm fibroin-like protein and caddisworm fibroin-like proteins, mirroring detected protein abundance as well as the characteristic motifs. Conservation of silkworm fibroin-like protein is limited in Apseudoidea, and only one of three caddisworm fibroin-like proteins (comp408) is conserved in this superfamily.

## Discussion

Here we present the comprehensive transcriptome sequencing and assembly of 23 tanaidaceans encompassing 14 families and 4 superfamilies of Tanaidacea. This is the largest data set of this underrepresented crustacean order to date. Comprehensive transcriptomic data will accelerate tanaidacean studies in evo-devo contexts, an otherwise untouched field. Here we focused on the silk use, but Tanaidacea harbors many other interesting topics. One example can be found in the convergent evolution of hermit-crab-like shape in Crustacea. One tanaidacean group shows striking morphological adaptations for gastropod-shell use (e.g., a twisted body) and utilizes empty gastropod shells as mobile shelters in the same fashion that phylogenetically distant decapods (= hermit crabs) do (Bird and Webber 2015; Kakui 2019), providing us with the opportunity to investigate which changes are general or group specific in the evolution of shell-inhabiting lifestyles in crustaceans. Another is the high diversity of sexual systems in Tanaidacea. Tanaidacea is the only taxon in Malacostraca, the most species-rich class in Crustacea, that contains simultaneous hermaphroditic species having separate male and female reproductive gonads (not forming an ovotestes; cf. Bauer and Holt 1998; Kakui and Hiruta 2013), which will give many hints toward understanding how the simultaneous hermaphroditic condition evolved and is maintained in Crustacea. Tanaidaceans also show variable morphology of eyes, and this feature was confirmed to also possess a molecular component, through the lack of short wavelength opsins in pigment-less and eye-less species (supplementary text, Supplementary Material online).

Through comprehensive phylogenetic analysis, all four superfamilies were recovered as fully supported monophyletic groups in the present tree, although the monophyletic condition of Apseudoidea was questioned relative to prior 18S-rRNA based analyses (Kakui et al. 2011). The relationships amongst the four superfamilies were identical to those in Kakui et al. (2011). Among five families containing two or more species used in this analysis, Akanthophoreidae was not recovered as a strongly supported monophyletic group. The nodal support for the clade comprised three akanthophoreid, one tanaellid, and one anarthrurid species was low (bootstrap value = 0.70; partitioned bootstrap value = 0.70; posterior probability = 1), suggesting the need for a more comprehensive analysis using more paratanaoidean groups and the reassessment of the family-level framework.

The use of silk distinguishes tanaidaceans from most other crustaceans, and through a multiomics approach combining comprehensive transcriptomics as well as silk proteomics from *Z. ezoensis*, we have identified two families of tanaidacean silks. The most abundant protein in the silk, comp9210, contained repetitive motifs similar to silkworm silk, whereas the second most abundant group (comp1, comp300, and comp408) shared conserved N-terminal sequences suggesting a homologous origin, and contained repetitive motifs resembling those of caddisworm silks. On the other hand, the N/C-terminal sequences showed no similarity to the fibroin genes of other species, suggesting an independent evolution of these silk genes in tanaidaceans. Although the repetitive motifs are similar to silkworm and caddisworm silks, tanaidacean silk proteins tend to be more hydrophilic compared with their analogs, representative of their unique adaptation to a marine environment. The tanaidoidean thoracic gland system harbors two secretory glands (tg1 and tg2), where tg1 is comparably larger than tg2 (Kaji et al. 2016). The existence of a pair of glands coincides with the two families of proteins identified, and the higher protein abundance of comp9210 is suggestive of its relationship with the larger tg1 gland, but a more detailed gland-specific expression analysis is necessary to fully determine such localizations.

These genes were found to be conserved throughout tanaidaceans, though some species lack expression. Among seven paratanaoidean families included in this study, Akanthophoreidae and Heterotanoididae lack members with previous records of tube-use (Kakui 2021). In all species in the two families (*Akanthophoreus* sp., *Chauliopleona* cf. *sinusa*, and *Parakanthophoreus* sp.; *Heterotanoides* sp.), the gene expression of the caddisworm fibroin-like proteins was not detected and that of the silkworm fibroin-like protein was low level or not detected, which may imply that they have actually adopted a silk-free mode of life, such as burrowing or subsistence in the epibenthos. However, even in the well-known tube-dwelling genus *Tanaella* (cf. Kakui 2021), whilst the gene expression of silkworm fibroin-like silk is moderately high, expression of caddisworm fibroin-like proteins was not detected. Likewise, *Neotanais*, which is epifaunal and lacks tube use, shows low, but detectable expression of comp9210 and comp408. More detailed study of inner morphology and gland-specific expression analyses of these species are required to fully understand these exceptions.

Three currently known gland systems related to tube constructions in Tanaidacea, namely thoracic-gland, pereopodal-gland, and pleotelsonal-gland systems (hereafter, Th-, Pe-, and Pl-systems), differ in various ways. In the Th-system found in Paratanaoidea and Tanaidoidea, silk is made by mixing secretions from two types of glands (Kaji et al. 2016); in the Pe-system in *Phoxokalliapseudes tomiokaensis* (Apseudoidea: Kalliapseudidae) and the Pl-system in *Parapseudes algicola* (Apseudoidea: Parapseudidae), there is no evidence of mixing secretions from multiple types of glands to make silk (Kakui and Hiruta 2014, 2017). Tubes made by the Th- and Pe-systems are stout enough to be picked up by forceps whereas those made by the Pl-system are not, indicating that the components of the tubes are different among systems. The gene expression patterns in Pe- and Pl-systems are similar to *Carpoapseudes spinigena*, a burrower (Kakui et al. 2020), indicating that the silk-related genes in tube dwellers bearing Pe- and Pl-systems may not be detected in our analysis and may greatly differ from those in tube dwellers bearing the Th-system. Of the three paralogs of caddisworm fibroin-like proteins, only comp408 is conserved in Apseudoidea, suggesting duplication of these genes in the common ancestor of Paratanaoidea and Tanaidoidea, following the divergence of Apseudoidea. Amongst four tanaidids, only *Arctotanais alascensis* lacks the expression of comp300. We observed that this species constructs stouter tubes than those of the other tanaidids (data not shown). The lack of comp300 in *A. alascensis* may therefore be related to the above difference in the tube feature.

## Materials and Methods

### Sampling of Tanaidaceans, Mode of Life Investigation, and Silk Collection

We collected 23 tanaidacean and one isopod species for transcriptomic analyses at various localities presented in supplementary table S1, Supplementary Material online. Fresh animals were fixed in RNAlater Stabilization Solution (Invitrogen) at 4 or −20 °C for 1 day, then kept at −20 °C for several days (only in some specimens due to facility limitation), and finally stored at −80 °C. Species identification of each specimen was made by observation of the specimen before fixation and of other specimens collected at the same locality and judged as conspecific (if available).

To check their mode of life, living individuals of nine tanaidacean species (*Apseudes* sp., *Paradoxapseudes littoralis*, *Phoxokalliapseudes tomiokaensis*, *Apseudomorpha* sp., *Parapseudes algicola*, and four tanaidid species) and *Asellus hilgendorfii* were reared at 4, 21, or 25 °C for weeks. The mode of *Carpoapseudes spinigena* was presented by Kakui et al. (2020). The mode of *Pseudosphyrapus quintolongus* was inferred based on the observed mode in the congener, *P. malyutinae* (Kakui K. unpublished data). The mode of the other species was inferred based on the literature regarding confamilial or congeneric species (cf. Kakui 2021).

Silk of *Z.ezoensis* was collected as follows. Around 80 individuals and several pieces of mesh (425-μm mesh opening) were put in a petri dish filled with 0.20-μm filtered seawater in a dark condition at 14 °C. The animals were fed every 3 days with porphyrized dry feed for crayfish (JAN code 4971618829092; Kyorin). The day after feeding, all individuals were transferred to a new dish filled with 0.45-μm filtered seawater twice, and finally to a new dish filled with 0.20-μm filtered seawater in the above condition (“cleaning”); new mesh pieces were put in the dish. The day after cleaning, tubes made by tanaidaceans were picked up with fine forceps, put in a 1.5-ml tube filled with 0.20-μm filtered seawater, and stored at 4 °C. Five samples were made.

### Transcriptome Sequencing and Assembly

Sample preservation, RNA extraction, sequencing, and assembly were conducted based on methods previously described for spiders (Kono et al. 2016), with some modifications. Briefly, a single specimen of each tanaidacean was preserved in RNAlater Stabilization Solution, then stored at −80 °C. RNA was extracted using a Direct-zol RNA Microprep Kit (Zymo Research). The Illumina library was prepared using the KAPA RNA HyperPrep Kit (KAPA Biosystems) targeting 300-bp fragments, but in order to amplify larger silk-related transcripts, PCR extension time was increased to 3 min per cycle. Extracted RNA was below minimum requirement for KAPA RNA HyperPrep Kit for samples R05 *Apseudomorpha* sp., R06 *Paradoxapseudes littoralis*, and R24 *Heterotanoides* sp., and therefore these samples were prepared using SMART-Seq v4 Ultra Low Input RNA Kit for Sequencing (Clonetech) also with increased PCR extension time, followed by KAPA HyperPlus Kit (KAPA Biosystems) fragmentation and cDNA library preparation. The sequence library was then sequenced on a NextSeq 500 (Illumina) with 300 cycles of high-output mode as paired-end reads. Sequences were base called and demultiplexed, and adaptor sequences were removed with bcls2fastq v.2 software (Illumina).

A total of 20 ng of *Z.ezoensis* extracted RNA was further utilized for long read sequencing. The mRNAs were amplified using SMART-Seq v4 Ultra Low Input RNA Kit for Sequencing for 12 PCR cycles, and following purification with 2X AMPure XP (Beckman Coulter), the cDNA was further amplified using ISPCR primer (5′-AAGCAGTGGTATCAACGCAGAGT-3′) for 12 cycles with SeqAmp DNA polymerase (Takara Bio) to eliminate blocked DNA ends. After 0.45X AMPure XP purification to filter out short transcripts, the cDNA sequencing library was prepared using Ligation Sequencing Kit (SQK-LSK109; Oxford Nanopore Technologies) with Short Fragment Buffer protocol. Resulting cDNA library was sequenced with R9.4.1 flow cell (FLO-MIN106) on a GridION device (Oxford Nanopore Technologies). Basecalling was performed using MinKNOW and the high accuracy mode of Guppy basecaller (Oxford Nanopore Technologies).

Transcriptome assembly was performed using Bridger with default parameters using Illumina reads (Chang et al. 2015). In order to eliminate possible cross contaminations, transcripts with TPM (transcripts per million) value less than 1 and transcript ID over 30,000 were discarded from the following analyses. Assembly completeness was assessed using BUSCO v.3 (Seppey et al. 2019) with the Arthropoda data set through the gVolante server (Nishimura et al. 2017). One SMART-Seq amplified sample R05 *Apseudomorpha* sp. resulted in very low BUSCO completeness (66.2%), and this sample was omitted in further analyses.

### Phylogenetic Analysis

A BUSCO analysis using *Drosophila* as the model species was conducted on each tanaidacean genome (Simao et al. 2015). As the *Apseudomorpha* sp. showed less than 75% completeness, it was removed from further phylogenetic analysis. Then, complete, single-copy BUSCO sequences (as determined vs. the reference species *Drosophila*) that were present in 20 out of the 23 remaining taxa (in over 85% of the genomes), were then selected for use in constructing the phylogeny. This generated a multiple sequence alignment of 28 BUSCO genes. Each gene was aligned separately in MUSCLE (Edgar 2004), and the resultant alignments were concatenated using SequenceMatrix (Vaidya et al. 2011) to produce the analysis data set. A preliminary maximum likelihood phylogenetic tree was constructed using the WAG model in PhyML (Whelan and Goldman 2001; Guindon et al. 2010), and a final maximum likelihood analysis was then undertaken in IQTree under the model recommended by ModelFinder, LG+F+I+G4 (Nguyen et al. 2015; Kalyaanamoorthy et al. 2017), with 1,000 bootstrap replicates. Following this, an additional Partitioned maximum likelihood analysis was undertaken in order to account for variation in evolutionary rates amongst genes (Lanfear et al. 2012). Partition files were generated using catsequences 1.3 (Creevey and Weeks 2021) and partition models were determined using the greedy partition finding algorithm present in IQTree (Chernomor et al. 2016). Finally, a Bayesian phylogenetic tree was constructed using the GTR+G model in Phylobayes 4.1 (Lartillot and Philippe 2004, 2006; Lartillot et al. 2007). Convergence was assessed by comparing the maximum discrepancies observed over the bipartitions and effective sample size in bpcomp and tracecomp. For all analyses, two independent chains were run. A burnin of 50% of the sample size was used for all analyses, sampling every 50th tree following the burnin period.

### Proteome Analysis of the *Z. ezoensis* Silk

PBS-washed silk samples were immersed in 6 M guanidine-HCI buffer (pH 8.5) and protein extraction was performed by homogenization with BioMasher II (Nippi) and sonication with Bioruptor II (BM Equipment). The extracts containing 10 µg of protein were reacted with dithiothreitol for 30 min at 37 °C followed by iodoacetamide for 30 min at 37 °C in the dark. After 5 times dilution by 50 mM ammonium carbonate in distilled water, the samples were reacted with Lys-C for 3 h at 37 °C followed by trypsin for 16 h at 37 °C to digest proteins. The reaction was quenched by acidification with trifluoroacetic acid and the digested samples were desalted using SDB-XC-StageTips (Rappsilber et al. 2003) followed by drying under reduced pressure.

Each sample was dissolved with 0.1% formic acid (FA) and 2% acetonitrile (ACN) and analyzed with a nanoElute, and a timsTOF Pro (Bruker Daltonics). Two hundred nanograms of the digest was injected into a column, Aurora UHPLC column C18 1.6 µm (75 µm ID × 250 mm, IonOpticks), and separated by liner gradient elution with two mobile phases: (A) 0.1% FA in water and (B) 0.1% FA in ACN, at the flow rate of 400 nl/min under the temperature at 50 °C. The mobile phase composition was changed as follows: (A) + (B) = 100%, (B) 2–17% (0–60 min), 17–25% (60–90 min), 25–37% (90–100 min), 37–80% (100–110 min), and 80% (110–120 min). Separated peptide was ionized at 1,600 V and analyzed by PASEF (Meier et al. 2018) scan. Briefly, the PASEF scan was performed at the ion mobility coefficients (1/K0) range from 0.6 to 1.6 V s/cm^2^ within the ramp time of 100 ms keeping the duty cycle at 100%. A MS scan was performed at the mass range from *m*/*z* 100 to 1,700, followed by 10 PASEF-MS/MS scans per cycle. Precursor ions was selected from top 12 intense ions in a TIMS-MS survey scan (precursor ion charge: 0–5, intensity threshold: 2,500, target intensity: 20,000). In addition, a polygon filter was applied to the *m*/*z* and ion mobility plane to select most likely representing peptide precursors without singly charged ions. Collision-induced dessociation was performed by the default settings (isolation width: 2 Th at *m*/*z* 700 and 3 Th at *m*/*z* 800, collision energy: 20 eV at 1/k0 0.6 V s/cm^2^ and 59 eV at 1/k0 1.6 V s/cm^2^).

LC-MS data were analyzed using PEAKS studio X+ (Bioinfomatics Solutions) (Zhang et al. 2012) with the following conditions. Briefly, de novo sequencing and database search were performed with the error tolerance of 20 ppm for precursor ions and 0.05 Da for fragment ions. Enzyme was set to trypsin, and up to two missed cleavages was allowed. Carbamidomethylation at cysteine residue was set as a fixed modification. N-acetylation at protein N-term and oxidation at methionine residue were set as variable modifications allowing for up to three positions per peptide. Protein sequence database (383,8921 entries) was created from transcriptome assembly data and used for identification. MaxQuant (version 1.6.10.43) (Cox and Mann 2008) contaminants database (245 entries, major experimental contaminants) was used as a contaminant database. Criteria of identification was set to less than 1% FDR at the peptide-spectrum match level. Feature area of each identified peptide ion was calculated automatically with PEAKS software algorithm. The intensity-based absolute quantification value (Schwanhausser et al. 2011) of each identified protein was calculated from the feature area values.

### Screening of Silk Genes and Their Conservation Analysis

PEAKS searches did not immediately result in silk-related genes, so the silk genes were screened from the proteome analysis with the following criteria: 1) lack of similarity to proteins in UniProt (BlastP *e*-value < 1e-15), 2) more than two peptide fragments identified from the protein, 3) consistent detection among replicates (normalized intensities have normalized standard deviation of *Z* < 1), and 4) repetitive units comprise at least 10% of the sequence as identified by the XSTREAM web service (Newman and Cooper 2007). Nanopore reads were searched using the putative silk transcripts, and the longest reads with the highest sequence identity within the Illumina transcriptome assembly were used to obtain the full-length sequence. Extracted nanopore reads corresponding to the putative silk transcripts were error corrected using proovread with the Illumina reads (Hackl et al. 2014). Using the full-length gene sequences as well as their translated amino acid sequences, conservation was analyzed by BlastP and TBlastN searches among the 23 transcriptome assemblies obtained in this work with *e*-value thresholds of 1e-15 and complexity filtering turned off. Expression levels of transcripts were quantified with Kallisto v.0.44 (Bray et al. 2016). The Kyte–Doolitle hydropathy plot was computed using the N-terminal 1,000 amino acids on the ProtScale server (Gasteiger et al. 2005).

## Supplementary Material

Supplementary data are available at *Genome Biology and Evolution* online.

## Supplementary Material

evab281_Supplementary_Data
