# Supplementary material for: Comprehensive Transcriptome Sequencing of Tanaidacea with Proteomic Evidences for Their Silk
Source: Genome Biol Evol. 2021 Dec 14;13(12):evab281. doi: 10.1093/gbe/evab281 (PMC8715525; doi:10.1093/gbe/evab281)
Supplement: evab281_Supplementary_Data [file evab281_supplementary_data.zip › SupplementalText_KK211008_clean.docx]

**Supplemental Information**

**Analysis of opsins in tanaidaceans.**

New opsin genes were identified from the available tanaidaceans using BLAST (e-value = 10e-7) (Camacho, et al. 2009) against a dataset consisting of previously published opsins from a broad range of functional opsin families (Fleming, et al. 2020). The identity of the recovered sequences was then confirmed using a further BLAST search against the entire nr database (e-value= 10e-20) to ensure that the sequences recovered could not be assigned to other groups of GPCRs. The recovered sequences were then compared to the Prosite consensus sequence to confirm their identity as opsins. Of the 87 recovered sequences, 69 possess the retinal binding domain (RBD) and opsin consensus sequence as established by Prosite. Of the remaining 18 sequences, 1 possesses the retinal binding domain on its 7TM helix but differs from the current opsin consensus sequence: however, this sequence possesses a close resemblance to an existing opsin-5: that of *Penaeus vannamei* (e value: 2e-39, 51% positives, 13% gap) and does not resemble any non-opsin sequences. As such, it was identified as a neuropsin, which was confirmed in the phylogenetic analysis. The remaining 17 sequences all closely resembled opsins (<10e-35 vs. nr) but lack the 7^th^ transmembrane and retinal binding domain. These were determined to be partial sequences (S Table 2).

The new opsins were added to an alignment of previously published opsins based on the dataset used in Fleming et al. (2020), and aligned in MUSCLE (Edgar 2004). The subsequent multiple sequence alignment was then analysed in IQTree under the model recommended by ModelFinder, LG+F+I+G4 (Kalyaanamoorthy, et al. 2017; Nguyen, et al. 2015) with 1000 Bootstrap replicates.

Our analyses on opsins, a group of light-sensitive proteins including those involved in vision, found evidence of opsins in Tanaidacea from all three major families – the rhabdomeric, ciliary and group 4 opsins: the latter of which comprises the neuropsins, RGR opsins, Go-opsins and peropsins (see Figure and Table below). The rhabdomeric opsins were the focus of our subsequent analyses, as across other Crustacea families, it is these that are used for visual processes (Cronin and Porter 2014). Our hypothesis was that the species that do not possess eyes will also show a reduction in or complete loss of rhabdomeric visual opsins. Broadly, this hypothesis was proven to be correct, with two exceptions. In *Siphonolabrum*, which is eyeless, we found a single low-wavelength-sensitive (LWS) opsin, and we found the same to be true in the pigment-less *Phoxokalliapseudes*. As these were both found in the genome, they may represent sequences that are present in the organism but not transcribed. However, the LWS opsins in both species possessed a retinal binding domain, suggesting that, were they to be transcribed, they would still be functional. This suggests that in these two clades, reduction in visual capabilities may have occurred relatively recently. Meanwhile, within the taxa that possess eyes, we still observed reduction of the visual opsin repertoire in some species, with none of the studied taxa possessing all five crustacean rhabdomeric opsin families. Some of this variation can potentially be explained through sampling and a lack of data, but a consistent lack of the short-wavelength-sensitivie (SWS) opsin is particularly notable. We found no evidence of this opsin outside of the Paraspeudidae and eye-possessing Apseudidae. This suggests that the SWS opsin may well have been lost in the shared ancestor of Paratanaoidea, Neotanaoidea and Tanaidoidea. Outside of the rhabdomeric opsins, we found irregular but firm presence of ciliary and the group 4 neuropsins in Tanaidacea. This is similar to the occurrence of these opsins within other members of the Crustacea and Insecta, where they perform non-visual functions (Henze and Oakley 2015). We did not find evidence of the additional opsin families proposed by (Ramirez, et al. 2016): bathyopsin, non-canonical r, chaopsin, or xenopsin. This might be an effect of basing our dataset on the (Fleming, et al. 2020) robust minimal opsin tree dataset, which recovered these groups as phylogenetic artefacts following application of the Canary Sequence Approach. However, even considering these additional, controversial new groups, there has yet to be evidence of their existence within the Ecdysozoa (Ramirez, et al. 2016), and as such it is unsurprising that no representatives of these groups were recovered.

Figure Phylogenetic tree of opsin genes found in Tanaidacea transcriptome assembly. This tree was constructed in IQTree under the LG+F+I+G4 model. 1000 Bootstraps were run, and bootstrap support values are marked on each node.

Table Conservation of opsin genes and eye morphology

**References:**

Camacho C, et al. 2009. BLAST+: architecture and applications. BMC Bioinformatics 10: 421. doi: 10.1186/1471-2105-10-421

Cronin TW, Porter ML. 2014. The evolution of invertebrate photopigments and photoreceptors. In Evolution of visual and non-visual pigments. In: Hunt DM, Hankins MW, Collin SP, Marshall NJ, editors. Evolution of visual and non-visual pigments. Boston: Springer. p. 105-135.

Edgar RC 2004. MUSCLE: multiple sequence alignment with high accuracy and high throughput. Nucleic Acids Res 32: 1792-1797. doi: 10.1093/nar/gkh340

Fleming JF, Feuda R, Roberts NW, Pisani D 2020. A Novel Approach to Investigate the Effect of Tree Reconstruction Artifacts in Single-Gene Analysis Clarifies Opsin Evolution in Nonbilaterian Metazoans. Genome Biol Evol 12: 3906-3916. doi: 10.1093/gbe/evaa015

Henze MJ, Oakley TH 2015. The Dynamic Evolutionary History of Pancrustacean Eyes and Opsins. Integr Comp Biol 55: 830-842. doi: 10.1093/icb/icv100

Kalyaanamoorthy S, Minh BQ, Wong TKF, von Haeseler A, Jermiin LS 2017. ModelFinder: fast model selection for accurate phylogenetic estimates. Nat Methods 14: 587-589. doi: 10.1038/nmeth.4285

Nguyen LT, Schmidt HA, von Haeseler A, Minh BQ 2015. IQ-TREE: a fast and effective stochastic algorithm for estimating maximum-likelihood phylogenies. Mol Biol Evol 32: 268-274. doi: 10.1093/molbev/msu300

Ramirez MD, et al. 2016. The Last Common Ancestor of Most Bilaterian Animals Possessed at Least Nine Opsins. Genome Biol Evol 8: 3640-3652. doi: 10.1093/gbe/evw248
