# Supplementary material for: Comprehensive Transcriptome Sequencing of Tanaidacea with Proteomic Evidences for Their Silk
Source: Genome Biol Evol. 2021 Dec 14;13(12):evab281. doi: 10.1093/gbe/evab281 (PMC8715525; doi:10.1093/gbe/evab281)
Supplement: evab281_Supplementary_Data [file evab281_supplementary_data.zip › Supplementary information_210412.docx]

Figure S1 Phylogenetic tree of Tanaidacea sequenced in this work constructed with Bayesian method with 32 BUSCO genes. The tree was constructed in Phylobayes under the GTR+G model. Posterior node support values are reported at each node.

Table S1 Collection information for specimens used in this study. KK, Keiichi Kakui

Supplementary Text Analysis of opsin in tanaidaceans.
