# Supplementary figures and images for: Comprehensive Transcriptome Sequencing of Tanaidacea with Proteomic Evidences for Their Silk
Source: Genome Biol Evol. 2021 Dec 14;13(12):evab281. doi: 10.1093/gbe/evab281 (PMC8715525; doi:10.1093/gbe/evab281)

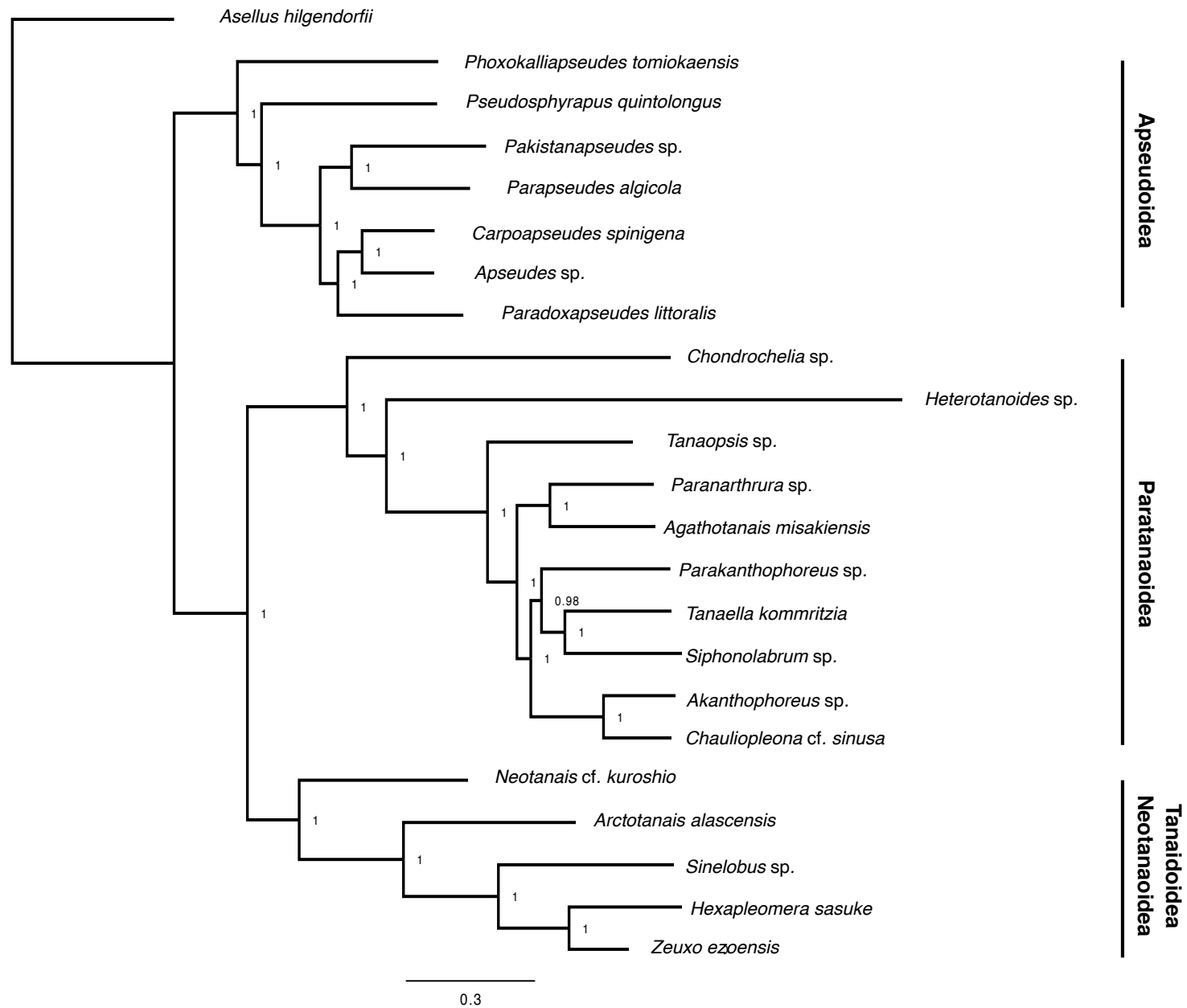

Figure S1 Bayesian tree

Supplement: evab281_Supplementary_Data [file evab281_supplementary_data.zip › supplementaryfigs.pdf]
